# Comparative transcriptomic analysis reveals the significant pleiotropic regulatory effects of LmbU on lincomycin biosynthesis

**DOI:** 10.1186/s12934-020-01298-0

**Published:** 2020-02-12

**Authors:** Chun-Yan Lin, Ai-Ping Pang, Yue Zhang, Jianjun Qiao, Guang-Rong Zhao

**Affiliations:** 1 Frontier Science Center for Synthetic Biology and Key Laboratory of Systems Bioengineering (Ministry of Education), School of Chemical Engineering and Technology, Tianjin University, Yaguan Road 135, Jinnan District, Tianjin, 300350 China; 2 SynBio Research Platform, Collaborative Innovation Centre of Chemical Science and Engineering (Tianjin), Tianjin University, Yaguan Road 135, Jinnan District, Tianjin, 300350 China; 3 Present Address: State Key Laboratory of Bioelectronics, School of Biological Science and Medical Engineering, Southeast University, Nanjing, 210096 China; 4 Present Address: Tianjin Institute of Industrial Biotechnology, Chinese Academy of Sciences, Tianjin, 300308 China

**Keywords:** *Streptomyces lincolnensis*, Lincomycin, LmbU, Transcriptional regulator, Pleiotropic regulation

## Abstract

**Background:**

Lincomycin, produced by *Streptomyces lincolnensis*, is a lincosamide antibiotic and widely used for the treatment of the infective diseases caused by Gram-positive bacteria. The mechanisms of lincomycin biosynthesis have been deeply explored in recent years. However, the regulatory effects of LmbU that is a transcriptional regulator in lincomycin biosynthetic (*lmb*) gene cluster have not been fully addressed.

**Results:**

LmbU was used to search for homologous LmbU (LmbU-like) proteins in the genomes of actinobacteria, and the results showed that LmbU-like proteins are highly distributed regulators in the biosynthetic gene clusters (BGCs) of secondary metabolites or/and out of the BGCs in actinomycetes. The overexpression, inactivation and complementation of the *lmbU* gene indicated that LmbU positively controls lincomycin biosynthesis in *S. lincolnensis*. Comparative transcriptomic analysis further revealed that LmbU activates the 28 *lmb* genes at whole *lmb* cluster manner. Furthermore, LmbU represses the transcription of the non-*lmb* gene *hpdA* in the biosynthesis of l-tyrosine, the precursor of lincomycin. LmbU up-regulates nineteen non-*lmb* genes, which would be involved in multi-drug flux to self-resistance, nitrate and sugar transmembrane transport and utilization, and redox metabolisms.

**Conclusions:**

LmbU is a significant pleiotropic transcriptional regulator in lincomycin biosynthesis by entirely activating the *lmb* cluster and regulating the non-*lmb* genes in *Streptomyces lincolnensis*. Our results first revealed the pleiotropic regulatory function of LmbU, and shed new light on the transcriptional effects of LmbU-like family proteins on antibiotic biosynthesis in actinomycetes.

## Background

Streptomycetes are famous for the ability to produce a great variety of valuable secondary metabolites, which have pharmacological activities such as antibacteria, antifungi, antiparasites, anticancer, and immunosuppression [1]. Genes encoding for biosynthesis of secondary metabolites are usually located together into the biosynthetic gene clusters (BGCs) in streptomycetes. For timely and coordinated expression, BGCs are under strict controls at the transcription level of intertwined regulation by pleiotropic regulators and pathway specific regulators [2]. The pathway specific regulators encoded by genes in BGCs of secondary metabolites activate or repress the expression of the BGCs for the biosynthesis of corresponding products. And the pleiotropic regulators associate various aspects involved in primary and secondary metabolisms, and the morphology of sporulation and pigmentation.

**Fig. 1:**
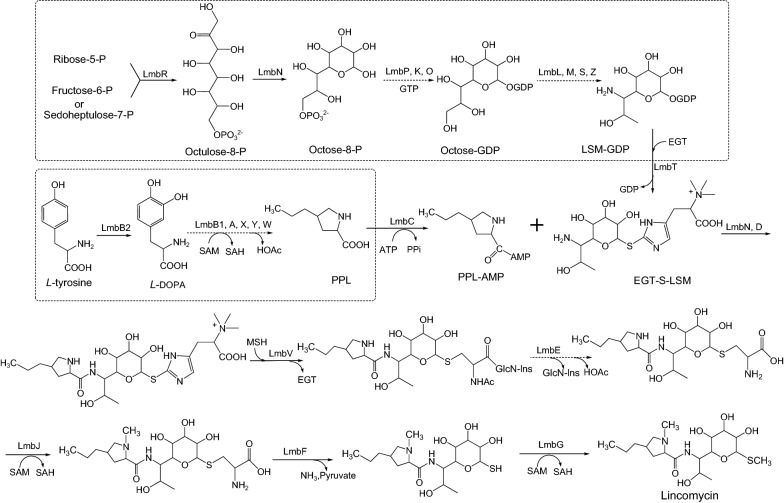
Putative lincomycin biosynthetic pathway in *Streptomyces lincolnensis*. LSM: lincosamide; EGT: ergothioneine; PPL: 4-propyl-l-proline; SAM: *S*-adenosylmethionine; SAH: *S*-adenosylhomocysteine; MSH: mycothiol; GlcN-Ins: 1-*O*-glucosamine-d-*myo*-inositol

Lincomycin, produced by *Streptomyces lincolnensi*s, is a lincosamide antibiotic used for the treatment of the infective diseases caused by Gram-positive bacteria [3]. The lincomycin biosynthetic (*lmb*) gene cluster has been partially characterized in *S. lincolnensis* [4, 5]. And the genomes of *S. lincolnensis* NRRL 2936 and *S. lincolnensis* LC-G have been sequenced up to date [6, 7]. The *lmb* cluster harbors three resistance genes (*lmrA*, *lmbrB* and *lmrC*) and twenty-six open reading frames (ORFs) with putative biosynthetic and regulatory functions. The biosynthetic pathway of lincomycin (Fig. 1) has been deeply explored in recent years. Lincomycin consists of a methylthiolincosaminide (MTL) moiety and an N-methylated 4-propyl-l-proline (PPL) moiety. The moiety MTL originates from a GDP-activated C8 sugar lincosamide (LSM), which is synthesized from the condensation of C5 donor α-d-ribose-5-phosphate and C3 donor fructose-6-phosphate (or d-sedoheptulose-7-phosphate) by LmbR and LmbN [8]. The biosynthesis of PPL begins with hydroxylation of l-tyrosine catalyzed by LmbB2 [9]. The ergothioneine (EGT) S-conjugated LSM and the ATP-activated PPL are assembled together by the condensation enzymes [10–14]. Finally, lincomycin is synthesized after modifications of thiols exchange, N-methylation, C–S bond cleavage and S-methylation [15, 16].

For transcriptional regulation of lincomycin biosynthesis, the pleiotropic regulators have been reported in previous studies. The orphan response regulator GlnR showed to enhance lincomycin biosynthesis by up-regulating the transcription of nitrate-specific ABC transporter and nitrate assimilation genes, and lincomycin exporter gene *lmrA* [17]. The TetR family regulator SLCG_2919 was identified to negatively regulate the transcription of the *lmb* cluster and its adjacent gene *SLCG_2920*, which encodes an ATP/GTP-binding protein associated to resistance against lincomycin [18]. The *bldA* gene encoding the tRNA with rare leucine codon UUA regulated morphological differentiation and lincomycin biosynthesis via controlling the translation of TTA-containing genes including *lmbB2* and *lmbU* in *S. lincolnensis* [19]. The developmental regulator BldD played a positive regulation role in both the sporulation of *S. lincolnensis* and the transcription of the partial *lmb* genes [20]. The A-factor AdpA was involved in the cascade regulation of lincomycin biosynthesis by activating some *lmb* genes and the *bldA* gene [21].

When the *lmb* cluster was firstly cloned, the *lmbU* gene was presumed to be a pathway specific regulatory gene [4]. Until recently, LmbU was partially characterized as a positive transcriptional regulator involved in lincomycin biosynthesis by its inactivation and overexpression experiments [6]. LmbU could activate several *lmb* genes by binding to DNA sites of the promoter regions through its helix-turn-helix motif [22]. However, the in vivo regulatory effects of LmbU on lincomycin biosynthesis have not been fully understood. On the other hand, several homologous LmbU-like proteins have been reported as pathway specific regulators in BGCs of novobiocin [23, 24], himastatin [25], hormaomycin [26] and actinomycin D [27]. SACE_5599 of *Saccharopolyspora erythraea,* a LmbU-like protein out of the erythromycin BGC, cross-regulates erythromycin production and the morphological differentiation [28]. Furthermore, SACE_5599 could regulate lincomycin biosynthesis by directly binding to promoters of *lmbA* and *lmbW* in vitro acting similarly to LmbU [6].

In this study, to comprehensively understand the regulatory effects of LmbU, we first conducted the bioinformatic analysis which showed that LmbU homologous proteins were universally distributed in actinomycetes and belong to a novel regulator family. Then, we constructed the *lmbU* deletion strain to perform transcriptome sequencing. Comparative transcriptomic analysis showed LmbU is a pleiotropic regulator. It positively regulates the transcription of the 28 *lmb* genes at whole *lmb* cluster manner. Moreover, LmbU positively regulates nineteen non-*lmb* (out of *lmb*) genes and negatively regulates one non-*lmb* gene in *S. lincolnensis* genome.

## Results

### In silico analysis of the putative pleiotropic regulatory function of LmbU

**Fig. 2:**
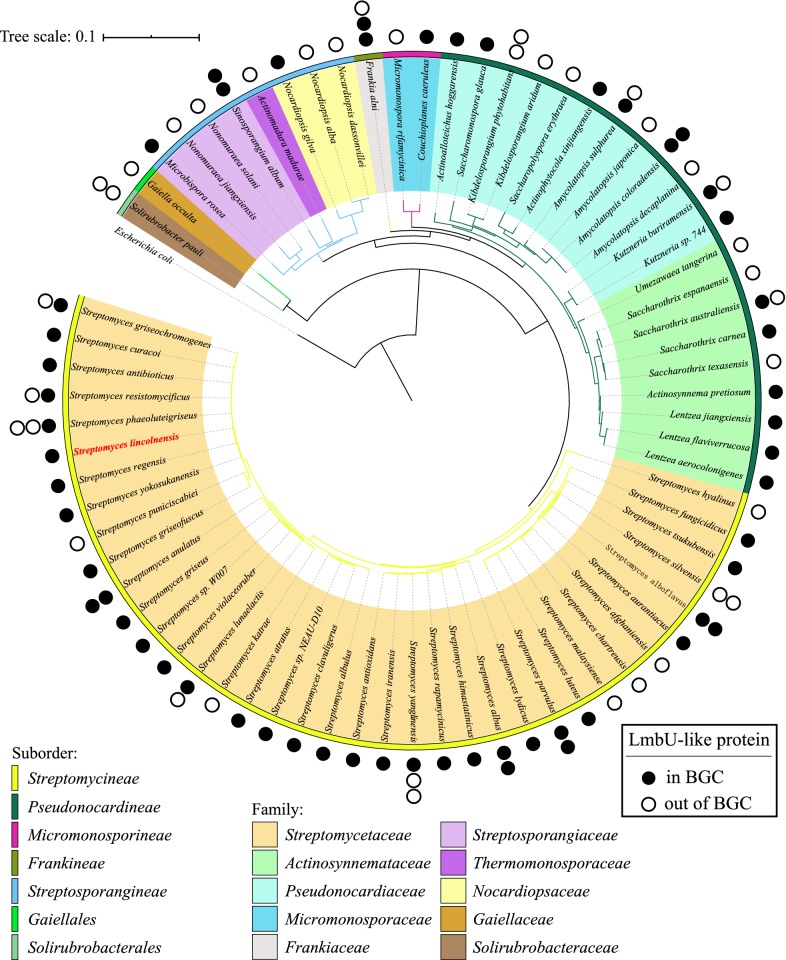
Phylogenetic analyses of actinomycetes with LmbU-like proteins. Rooted Neighbor-joining phylogeny of 72 actinomycetes based on their 16S sRNA sequences. The families separated in ten groups are represented by different color shaded areas. The filled circular shapes represent LmbU-like proteins in the BGCs and the open circular shapes represent out of the BGCs. The GenBank accession numbers of 16S sRNA genes and 93 LmbU-like proteins are listed in Additional file 1: Table S1

LmbU-like proteins with similarity over 49% were collected by BLASTP search against NCBI database using LmbU protein as query sequence. Then 93 LmbU-like proteins were selected based on their complete flanking sequences, which were used to locate *lmbU*-like genes either in the BGCs or not. The 16S rRNA genes from strains hosting these LmbU-like proteins were used to construct a phylogenetic tree (Fig. 2). LmbU-like proteins distribute in 72 species of 10 families of actinomycetes. More than half species (38 of 72) belong to genus *Streptomyces*, and others (34 of 72) belong to 21 genera from families *Actinosynnemataceae*, *Pseudonocardiaceae*, *Micromonosporaceae*, *Frankiaceae*, *Streptosporangiaceae*, *Thermomonosporaceae*, *Nocardiopsaceae*, *Gaiellaceae* and *Solirubrobacteraceae*. Sequence analysis of upstream and downstream regions around the *lmbU*-like genes revealed that nearly two-thirds of *lmbU*-like genes are located in the BGCs (mainly nonribosomal peptide BGCs, or polyketide-nonribosomal peptide BGCs) with single copy, similar to *lmbU* in the *lmb* cluster of *S. lincolnensis.* Furthermore, several BGCs have two *lmbU*-like genes. For example, the actinomycin C BGC of *Streptomyces anulatus* contains *acmO* and *acmJ* [29]. One-third of *lmbU*-like genes are out of the BGCs, such as SACE_5599 is not in the erythromycin or any predicted BGC of *Saccharopolyspora erythraea*. It is interesting that some actinobacteria possess *lmbU*-like genes both in and out of the BGCs. For instance, *Streptomyces lunaelactis* strain MM109, a ferroverdin A (anticholesterol) producer, contains two *lmbU*-like genes, one in a predicted nonribosomal peptide BGC and another out of BGC [30]. Thus, LmbU-like proteins are highly conserved and spread widely in actinobacteria, and they might be a new regulator protein family with pleiotropic regulatory roles in the biosynthesis of secondary metabolites and other biological process in actinomycetes.

### Functionality of *lmbU* regulating lincomycin biosynthesis

**Fig. 3:**
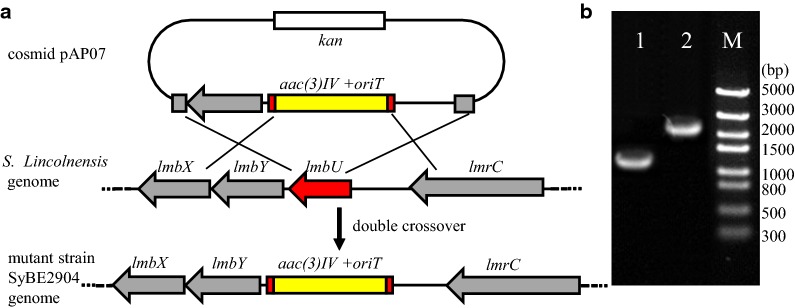
Deletion of the *lmbU* gene. **a** Schematic representation of the in-frame deletion of *lmbU*. A 617-bp region of *lmbU* was replaced by the 1370-bp *aac(3)IV*-*oriT* fragment through double crossover. **b** Confirmation of the *lmbU* deletion strain SyBE2904 by PCR amplification. Lane 1: a 1370-bp product using the original SyBE2901 genomic DNA as a template; Lane 2: a 2123-bp product using the SyBE2904 genomic DNA as a template; M: DNA molecular weight standard

**Fig. 4:**
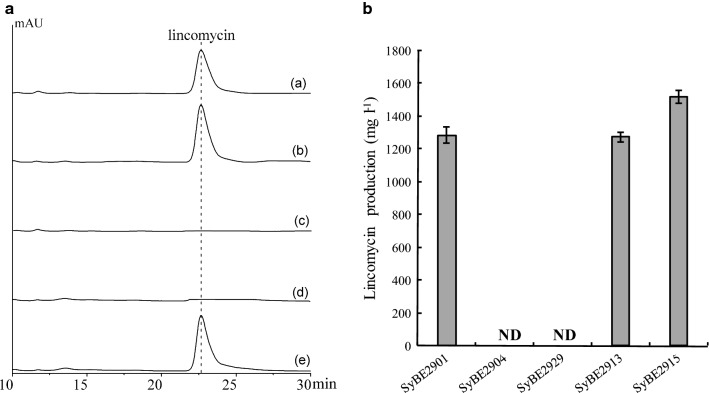
lincomycin production in the fermentation. **A** HPLC map of lincomycin: Standard lincomycin (a), Lincomycin produced by the original strain SyBE2901 (b), by the *lmbU* deletion strain SyBE2904 (c), by the *lmbU* complementation strain SyBE2929 (d), and by the *lmbUYX* complementation strain SyBE2913 (e). **B** lincomycin production in the fermentation of strains SyBE2901, SyBE2904, SyBE2929, SyBE2913, and SyBE2915. ND, not detected

To characterize the *lmbU* gene, the in-frame *lmbU* deletion strain SyBE2904 was constructed (Fig. 3a), which was confirmed by PCR (Fig. 3b), and further verified by DNA sequencing. The fermentation was conducted and lincomycin production in broth was measured by HPLC (Fig. 4A). As shown in Fig. 4B, compared to the original strain SyBE2901, the *lmbU* deletion strain SyBE2904 completely lost the ability to produce lincomycin during the fermentation, suggesting that inactivation of *lmbU* blocked the biosynthesis of lincomycin.

To complement the *lmbU* gene, three vectors (pHZ1358-U, pANT1201-U, and pLCY010-U) for the *lmbU* expression with different promoters and resistance genes (Table 1) were constructed and then introduced into the *lmbU* deletion strain SyBE2904, respectively. However, they all failed to resume lincomycin production. Analyzing the sequences of the *lmbU* gene and its downstream genes (*lmbY* and *lmbX*), the intergenic region between *lmbU* and *lmbY* is only 145-bp long, and the initiation codon of *lmbX* is overlapped with the termination codon of *lmbY,* which resulted in tetranucleotide sequence AUGA as stop-start codon. Thus, in-flame deletion of the *lmbU* gene might cause the polar effect on the expression of the *lmbY* and *lmbX* genes. The vector with fragment contains the *lmbU*-*lmbY*-*lmbX* genes under P_ermE*_ promoter was constructed and introduced into the *lmbU* deletion strain SyBE2904, and the resulting strain SyBE2913 restored the production of lincomycin (Fig. 4A). Moreover, the productivity of the *lmbU* overexpression strain SyBE2915 increased to 118.2% of the original titer of lincomycin (Fig. 4B). All results suggested that the *lmbU* gene encodes a positive regulator for activation of lincomycin biosynthesis.

**Table 1:** Strains and plasmids used in this study

Strain or plasmid	Characteristics	Reference or source
Strain		
*E. coli*		
DH5α	General cloning host	Invitrogen
BW25113/pIJ790	Host for λ-Red recombination	[47]
ET12567/pUZ8002	Donor strain for intergeneric conjugation	[48]
*S. lincolnensis*		
SyBE2901	Original strain for high lincomycin-producer, derived from ATCC25466	[46]
SyBE2904	SyBE2901 ∆*lmbU*	This study
SyBE2913	SyBE2904 with pLCY010-UYX	This study
SyBE2915	SyBE2901 with pLCY010-U	This study
SyBE2929	SyBE2904 with pLCY010-U	This study
SyBE2930	SyBE2904 with pHZ1358-U	This study
SyBE2931	SyBE2904 with pANT1201-U	This study
Plasmid		
SuperCos1	pUC ori, kan^r^, amp^r^	Novagen
pIJ773	Contains the *aac(3)IV* and *oriT* fragment	[47]
pUWL201apr	pUWL201 derivative, amp^r^, tsr^r^, apr^r^, *oriT*, carrying ermE* promoter	[49]
pHZ1358	Replicative, tsr^r^, *oriT*	[50]
pANT1201	Replicative, neo^r^, *oriT*, carrying *snpA* promoter	[51]
pLCY010	pUWL201 derivative, replicative, amp^r^, tsr^r^, hyg^r^, carrying ermE* promoter	[46]
pAP07	SuperCos1 derivative for deletion of *lmbU,* kan^r^, amp^r^, apr^r^	This study
pHZ1358-U	pHZ1358 with *lmbU*	This study
pANT1201-U	pANT1201 with *lmbU*	This study
pLCY010-U	pLCY010 with *lmbU*	This study
pLCY010-UYX	pLCY010 with *lmbUYX*	This study

### Organization of transcriptional units in the *lmb* cluster

**Fig. 5:**
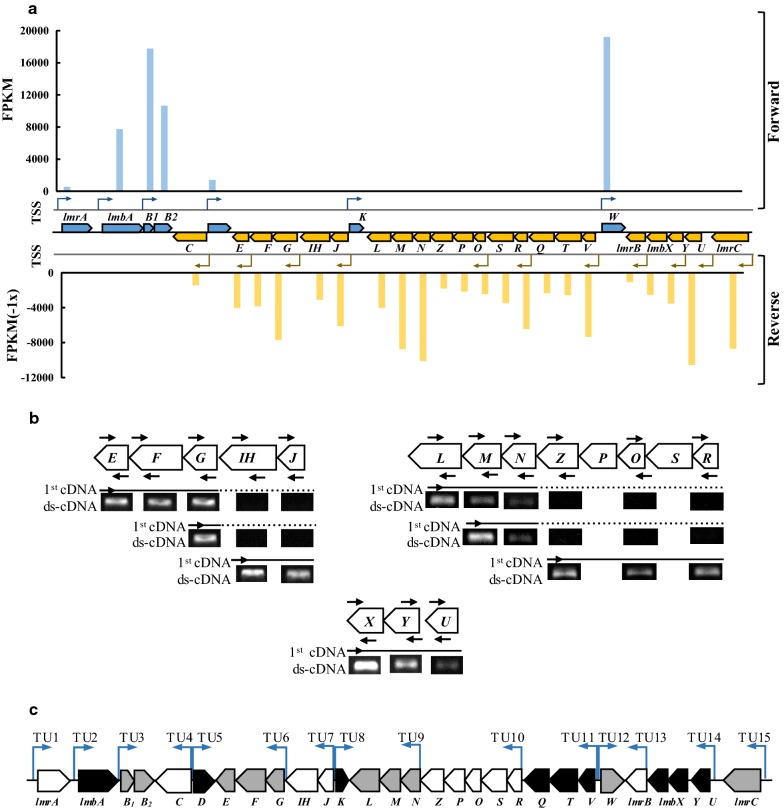
Identification of the transcription units of the *lmb* cluster. **a** Visualization of the predicted TTSs distribution in the *lmb* gene cluster and FPKM of each genes by ssRNA-Seq. The predicted TTSs are shown by bent arrows. Pointed boxes indicate the transcription directions of genes. The FPKM value columns of genes in sense strand are represented by blue, and those in antisense are represented by orange. **b** Detection of upstream transcripts by RT-PCR. The 1st cDNAs were prepared using antisense primers of the downstream genes, and the ds-cDNAs were amplified using each pair of primers (arrowheads) of the upstream genes. The analysis was carried out three times for each reaction, and the identity of each amplification product was authenticated by direct sequencing. **c** Transcription units of the *lmb* cluster. The vertical arrows connect the adjacent *lmb* genes and point to the direction of the gene transcriptions

The transcription patterns of the 29 ORFs in the *lmb* cluster were complicated. Of which, four genes (*lmbC*, *lmbD*, *lmbK*, and *lmbW*) were considered to be transcribed as the monocistronic units, as deduced from their chromosomal arrangements (Fig. 5a). The rest genes of the *lmb* cluster could be transcribed as polycistronic units. The contiguous genes overlapped coding sequences (usually GUGA or AUGA as stop–start codon) were considered to be transcribed and translated together, including *lmbB1*-*B2* (overlapping 4 bp), *lmbF*-*G* (overlapping 4 bp), *lmbIH*-*J* (overlapping 8 bp), *lmbL*-*M*–*N*-*Z*-*P*-*O* (overlapping 4 bp, respectively), *lmbS*-*R* (overlapping 4 bp), *lmbQ*-*T*-*V* (overlapping 4 bp, respectively), and *lmbY*-*X* (overlapping 4 bp) (Fig. 5a). Thus, there were at least 12 transcriptional units (TUs) for the 29 ORFs in the *lmb* cluster.

In order to identify TUs in the *lmb* cluster, strand specific RNA sequencing (ssRNA-Seq) was conducted from the original strain SyBE2901 at 3 day fermentation. The number of FPKM, fragments per kilobase of transcript sequence per millions base pairs sequenced, was used to estimate the expression level of each gene, and Rockhopper soft [31] was used to predict Transcription Start Site (TSS) paired with Transcription Termination Site (TTS) in the *lmb* cluster. The distribution of TTSs in the *lmb* cluster and the FPKM value of each gene were shown in Fig. 5a. According to the existence of contiguous overlapping reads, the distribution of TTSs and the degressive expression levels of following genes in one transcription unit, 17 TUs in the *lmb* cluster were preliminarily deduced.

For the subcluster *lmbAB1B2*, two TTSs were predicted at the upstreams of the *lmbA* and *lmbB1B2* genes, respectively, and FPKM of the *lmbB1* gene was much larger than that of the *lmbA* gene, demonstrating the *lmbA* gene and *lmbB1B2* gene were transcribed separately. The distributions of TTSs and TSSs showed three TUs (*lmbE*, *lmbG*-*F* and *lmbJ*-*IH*) in the subcluster *lmbJIHGFE*. However, the FPKMs of *lmbE* and *lmbG* were not significantly different, indicating that *lmbG*-*F*-*E* might be co-transcribed as one TU. In the case of the compact subcluster *lmbRSOPZNML*, FPKMs were degressive from *lmbV* to *lmbQ*, *lmbR* to *lmbZ*, and *lmbN* to *lmbL*, respectively. Although TSS was predicated in the upstream of *lmbO* instead of *lmbN*, the FPKM values of *lmbNML* were much larger than those of *lmbRSOPZ* genes in their upstreams, indicating the transcription of *lmbNML* was separated from those of *lmbRSOPZ*. Thus, the subcluster *lmbVTQRSOPZNML* was predicted to be transcribed into three TUs (*lmbV*-*T*-*Q*, *lmbR*-*S*-*O*-*P*-*Z* and *lmbN*-*M*-*L*). For the subcluster *lmbUYX*, although the FPKMs of *lmbU*, *lmbY,* and *lmbX* were degressive following transcriptional direction, one TSS was predicted in the upstreams of *lmbU* and *lmbY*, respectively, suggesting that *lmbU and lmbYX* might be transcribed as two TUs.

In order to eliminate the non-determinacy of TSS prediction and verify the co-transcription of the subclusters *lmbJIHGFE, lmbVTQRSOPZNML* and *lmbXYU*, reverse transcription polymerase chain reaction (RT-PCR) was further performed. The gene specific antisense primers listed in Additional file 1: Table S2 were used to synthesize the first strand DNA (1st cDNA), and then the upstream genes were amplified by PCR to detect whether the genes were cotranscribed or not. As shown in Fig. 5b, in the subcluster *lmbJIHGFE*, using the 1st DNA_lmbE_ (synthesized by antisense primer of *lmbE*) as template, the ds-cDNAs of the upstream genes *lmbF* and *lmbG* were detected, and the ds-cDNA of *lmbIH* was undetected. Furthermore, the ds-cDNA of *lmbJ* was detected using 1st DNA_lmbIH_ as template, indicating the transcription of the subcluster *lmbJIHGFE* was divided into two TUs (*lmbG*-*F*-*E* and *lmbJ*-*IH*). In the case of the subcluster *lmbRSOPZNML*, the ds-cDNA of *lmbN* was detected while that of *lmbZ* was undetected using the 1st DNA_lmbL_ as template, indicating the individual transcription of *lmbNML*. Meanwhile, the ds-cDNAs of *lmbR* and *lmbO* were detected using the 1st DNA_lmbZ_ as template, indicating the co-transcription from *lmbR* to *lmbZ* genes. These results confirmed the subcluster *lmbRSOPZNML* was transcribed as two TUs (*lmbN*-*M*-*L* and *lmbR*-*S*-*O*-*P*-*Z*). A similar result was observed between *lmbX* and *lmbU*, indicating the co-transcription of lmbX, lmbY, and lmbU, which was consistent with the complementation to the *lmbU* deletion by expression of the *lmbUYX*. Taking together, 15 transcription units of the *lmb* cluster were identified, including eight monocistrons (TU1, TU2, TU4, TU5, TU8, TU12, TU13, TU15), two bicistrons (TU3, TU7), four tricistron (TU6, TU9, TU11, TU14) and one pentacistron (TU10) (Fig. 5c).

### Comparison of transcriptomes between the original strain and the *lmbU* deletion strain

To obtain insight into the changes in gene expression levels between the original strain SyBE2901 and the *lmbU* deletion strain SyBE2904, RNA was isolated and subjected to whole-transcriptome sequencing via ssRNA-seq. Approximately 36.59 million 150 bp paired-end clean reads per sample were obtained after cleaning and checking the reads quality. Approximately 98% of clean reads were aligned uniquely to the *S. lincolnensis* genome. The expression level of each gene was calculated using FPKM. The correlation clustering among the two biological replicates of each sample was conducted based on the expression level of all genes. All biological replicates (two RNA samples for SyBE2901 and three RNA samples for SyBE2904) showed correlation coefficients over 0.9, indicating good reproducibility between biological replicates. To investigate transcriptional regulation effects of LmbU, the whole transcriptomes were compared.

**Fig. 6:**
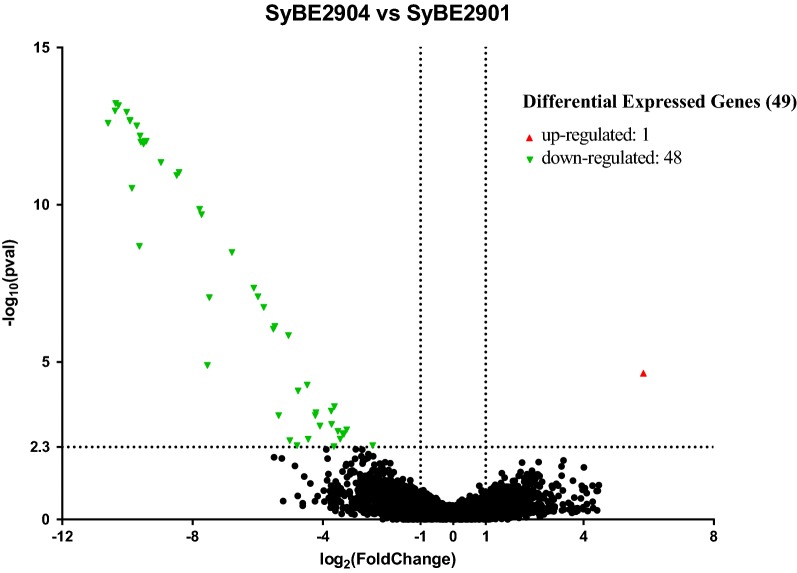
Volcano blots to show significant changes in gene expression between the original strain SyBE2901 and the *lmbU* deletion strain SyBE2904. Dispersion graph of the − log_10_(*p* value) (*y* axis) against the log_2_(fold change) (*x* axis) corresponding to the genes by their differential expression

Volcano plot was presented to identify genes with both high fold change and significance between the original strain SyBE2901 and the *lmbU* deletion strain SyBE2904 (Fig. 6). For high change, the foldchange of a gene between SyBE2904 and SyBE2901 should be > 1/2 and < 2, that means − 1 < log_2_(foldchange) < 1. For significance, the *p* value of a gene should be < 0.005, that means − log_10_(*p* value) > 2.3. Compared to the original strain SyBE2901, the transcription expressions of 49 genes were statistically different, of which 48 genes displayed decreased transcription levels, and only one gene displayed increased transcription level in the *lmbU* deletion strain SyBE2904. The results demonstrated that LmbU acted as a pleiotropic regulator for the positive or negative control expression of the genes including the *lmb* cluster and the non-*lmb* genes for lincomycin biosynthesis.

### LmbU upregulates the transcription of all genes in the *lmb* cluster

**Fig. 7:**
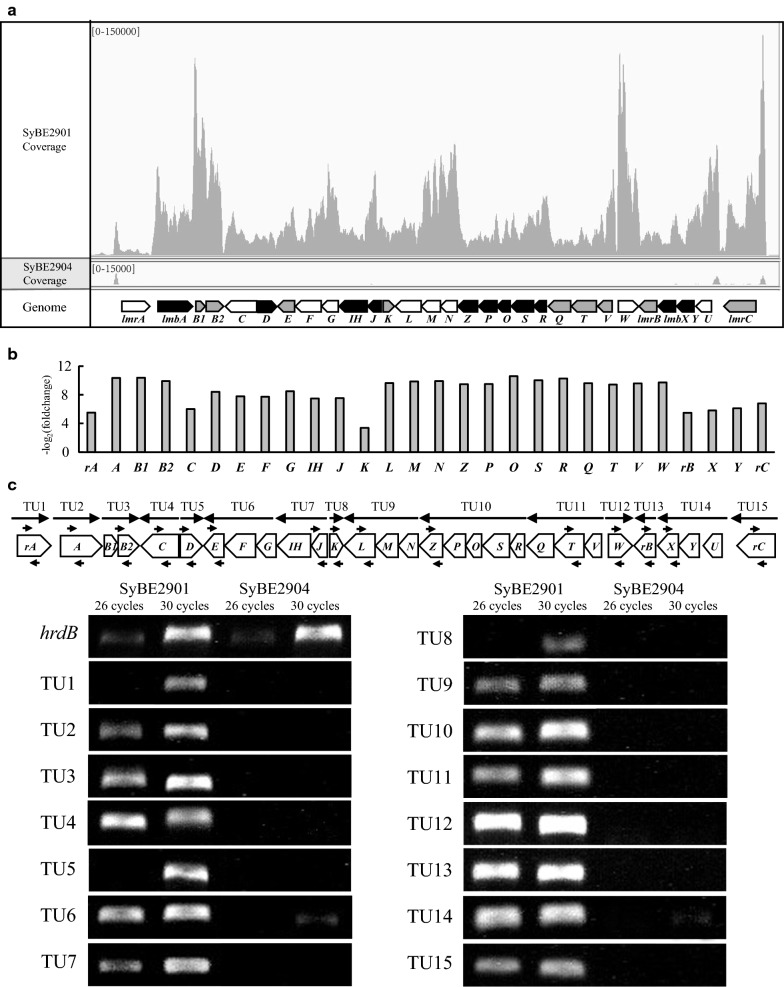
Difference transcriptional levels of the *lmb* gene cluster in the *lmbU* deletion strain SyBE2904 compared to original strain SyBE2901. **a** RNA-Seq maps. The different coverage of the RNA sequencing reads mapped to the *lmb* gene cluster of SyBE2901 and SyBE2904. **b** The − log_2_ fold change readcounts of the *lmb* genes between SyBE2901 and SyBE2904. **c** Semi-quantitative PCR to verify the results of RNA-Seq. One *lmb* gene in each TU was amplified using genomes of strains SyBE2901 and SyBE2904 as templates for 26 and 30 thermocycles, respectively. In each case the experiments were independently repeated three times

Of 49 differential expression genes observed from ssRNA-Seq results, 29 genes are the *lmb* genes encompassing the entire *lmb* cluster. Figure 7a showed that the reads from strain SyBE2901 mapping to the *lmb* cluster were extremely more than those from SyBE2904, which demonstrated that the significant change of the transcriptional levels of all 29 *lmb* genes between these two strains. No reads mapped to the *lmbU* gene showing the successful deletion of the *lmbU* in strain SyBE2904. When the *lmbU* gene was inactive in strain SyBE2904, the transcriptional levels of the rest 28 *lmb* genes were declined by 93.71% to 99.98% compared to SyBE2901. These variation multiples could be also expressed by the − log_2_ foldchange value (Fig. 7b). The genes in entire *lmb* cluster were barely transcribed in SyBE2904, which were consistent with the results of semi-quantitative PCR analysis (Fig. 7c). PCR products of TU1, TU5, and TU8 were detected for 30 thermocycles instead of 26 thermocycles in strain SyBE2901, indicating that those four TUs were transcribed at low levels in strain SyBE2901. In the case of the other twelve TUs (TU2, TU3, TU4, TU6, TU7, TU9, TU10, TU11, TU12, TU13, TU14, and TU15), PCR products were all strongly detected in strain SyBE2901 for both 26 and 30 thermocycles, indicating that those TUs were transcribed at high levels in strain SyBE2901. However, PCR products of 15 TUs were not detected in strain SyBE2904 for 26 and 30 thermocycles, respectively, indicating that the transcriptions of all 15 TUs were completely blocked by the deletion of the *lmbU* gene. Taking together, LmbU positively and strongly regulates the transcription of the 28 *lmb* genes in the *lmb* cluster.

### LmbU regulates transcription of non-*lmb* genes in *S. lincolnensis* genome

In addition to positive regulation on the expression of the whole *lmb* cluster, the results of RNA-Seq analysis showed that twenty non-*lmb* genes are regulated by LmbU (Table 2). Of which, eight genes were selected to perform semi-quantitative PCR analysis (Additional file 1: Fig. S1), and the results were consistent with those of ssRNA-Seq.

**Table 2:** List of differentially regulated non-*lmb* genes in the *lmbU* deletion strain compared to original strain

Gene_id	Readcount		log_2_ fold change	*p* value	Description	ID/SM (%)	Origin (protein accession number)	EC number
	SyBE2901	SyBE2904						
SLCG_3526	67.75	3879.47	5.84	2.28E−05	4-Hydroxyphenylpyruvate dioxygenase	94/98	*Streptomyces olindensis,* KDN74316.1	1.13.11.27
Transmembrane transporters (group 1)								
SLCG_3493	2228.6	402.1	− 2.47	0.0045383	MFS transporter, NarK/NasA family nitrate transporter	93/95	*Streptomyces canus*, KUN59611.1	
SLCG_6997	1262.37	68.17	− 4.21	0.0004083	MFS transporter, oxalate:formate antiporter family transporter	94/98	*Streptomyces indicus*, SDJ51645.1	
SLCG_7561	190.68	19.78	− 3.27	0.001436	MFS transporter, drug:H^+^ antiporter-2 (DHA2) family efflux transporter	71/81	*Streptomyces* sp. ST1015, WP_112483955.1	
SLCG_0089	26.2	0.81	− 5.01	0.0031263	Sugar ABC transporter substrate-binding protein	97/99	*Streptomyces mirabilis*, WP_052067725.1	
SLCG_7139	1957.42	146.97	− 3.74	0.0009585	ABC transporter permease	88/92	*Streptomyces africanus*, WP_086562194	
SLCG_7140	11211.76	501.86	− 4.48	5.38E−05	ATPase components of drug ABC transporters with duplicated ATPase domains	90/95	*Streptomyces caeruleatus*, KUO05737.1	
SLCG_7316	494.96	36.96	− 3.74	0.00036306	ABC-C family, MdlB, ATPase components of ABC-type multidrug transport system,	93/96	*Streptomyces resistomycificus*, KOG34263	
SLCG_1725	35.05	1.26	− 4.79	0.0045114	Drug/metabolite transporter (DMT) superfamily protein, threonine/homoserine efflux transporter RhtA	86/90	*Streptomyces* sp. MMG1533, KOU59304.1	
Oxidoreductase (group 2)								
SLCG_3218	352.14	20.68	− 4.09	0.0010757	NADPH-dependent FMN reductase, predicted flavoprotein	79/84	*Streptomyces viridochromogenes*, ELS54031.1	
SLCG_7376	1426.23	113.98	− 3.65	0.00025948	Succinate dehydrogenase cytochrome b subunit	95/97	*Streptomyces variegatus*, KJK34840.1	
SLCG_2976	475.18	25.3	− 4.23	0.00050136	oxidoreductase, zinc-binding dehydrogenase, possible enoyl reductase	85/90	*Streptomyces* sp. Go-475, AXE86817.1	1.6.5.5
SLCG_6211	743.9	18.18	− 5.35	0.00050014	Antibiotic biosynthesis monooxygenase	84/90	*Streptomyces viridochromogenes*, KMS69930.1	
SLCG_7612	883.35	32.55	− 4.76	8.28E−05	SgcJ/EcaC family oxidoreductase, nuclear transport factor 2 (NTF2-like) superfamily	88/93	*Streptomyces aureocirculatus*, WP_107055010.1	
Cellular metabolic enzyme (group 3)								
SLCG_0221	3436.88	103.23	− 5.06	1.44E−06	Carbonic anhydrase	92/97	*Streptomyces* sp. NEAU-C151, TLS40487.1	4.2.1.1
SLCG_1534	246.75	24.12	− 3.35	0.0019877	Epimerase	90/92	*Streptomyces fulvoviolaceus*, WP_030610101.1	
SLCG_6998	2683.63	242.47	− 3.47	0.0028291	Acetyl-CoA synthetase	95/97	*Streptomyces indicus*, SDJ51622.1	6.2.1.13
SLCG_7000	1580.04	228.61	− 2.79	0.0060806	IlvB, acetolactate synthase large subunit	99/99	*Streptomyces* sp. NL15-2K, GCB44424.1	2.2.1.6
Hypothetical protein (group 4)								
SLCG_0224	1575.31	135.29	− 3.54	0.0016004	Hypothetical protein	85/93	*Streptomyces* sp. NL15-2K, WP_124439655.1	
SLCG_1709	40.05	3.17	− 3.66	0.0048838	Hypothetical protein	85/92	*Streptomyces* sp. HGB0020, EPD63200.1	

*SLCG_3526* encodes a 4-hydroxyphenylpyruvate dioxygenase (HpdA), which is an Fe(II)-containing non-heme enzyme, and catalyzes the oxidation and decarboxylation of 4-hydroxyphenylpyruvate (precursor of l-tyrosine) to homogentisic acid [32]. The transcript of *hpdA* in the original strain was minor, while significantly increased after the *lmbU* deletion in strain SyBE2904, indicating that LmbU negatively regulated the *hpdA* expression and might be involved in the biosynthetic pathway of l-tyrosine via blocking the degradation of precursor 4-hydroxyphenylpyruvate.

In contrast to the *hpdA* gene, the nineteen non-*lmb* genes were transcriptionally decreased in strain SyBE2904 after the *lmbU* deletion, compared with the original strain, which indicated that LmbU positively regulates those genes. Based on biochemical and cellular functions, the nineteen non-*lmb* genes could be classified into four groups. Group 1 genes encode the transporters [33], including three major facilitator superfamily (MFS) transporters (SLCG_3493, SLCG_6997, and SLCG_7561), four ATP-binding cassette (ABC) superfamily transporters (SLCG_0089, SLCG_7139, SLCG_7140, and SLCG_7316), and a drug/metabolite transporter (DMT) superfamily protein (SLCG_1725). Group 2 genes encode the five redox proteins and enzymes, including a flavoprotein (SLCG_3218), a succinate dehydrogenase (SLCG_7376), an enoyl reductase (SLCG_2976), an antibiotic biosynthetic monooxygenase (SLCG_6211), and a SgcJ/EcaC family oxidoreductase (SLCG_7612). Group 3 genes encode cellular metabolic enzymes, including an epimerase (SLCG_1534), an acetyl-CoA synthetase (SLCG_6998), an acetolactate synthase (SLCG_7000), and a carbonic anhydrase (SLCG_0221). The last group 4 genes encode two hypothetical proteins (SLCG_0224 and SLCG_1709) without definite properties deduced from the NCBI database. To sum up, the results indicated LmbU would regulate the degradation, transport, oxidoreduction, and anabolism for lincomycin biosynthesis.

## Discussions

Homologous *lmbU*-like regulators are highly conserved proteins, and their genes widely co-exist in the BGCs of secondary metabolites or are distributed out of the BGCs, or both of them in actinomycetes which suggests that LmbU could be a new family of the transcription regulators, targeting the specific pathways or/and the other cellular metabolisms. Here, our comparative transcriptomics revealed that LmbU is a pleiotropic regulator via activating the transcription of the whole *lmb* cluster and regulating the transcription of non-*lmb* genes that scattered in the chromosome of *S. lincolnensis*, both involved in lincomycin biosynthesis.

The positive regulation of LmbU on the lincomycin biosynthesis was firstly confirmed by the *lmbU* deletion which abolished lincomycin production, and validated by the *lmbU* complementation and overexpression which restored and increased the titers of lincomycin, while no obvious morphology changes were observed after genetic manipulation in *S. lincolnensis*. It was consistent with the previous reports on the *lmbU* deletion and overexpression strains [6]. To obtain the insight on the regulatory properties of LmbU on lincomycin biosynthesis, analysis of comparative transcriptomes between the original strain and the *lmbU* deletion strain was performed in this study. Unexpectively, LmbU positively regulates all of the 28 *lmb* genes as the regulatory targets, including the structural genes encoding enzymes for catalyzing biosynthesis of lincomycin, and resistance genes (*lmrA*, *lmrB,* and *lmrC*) in the *lmb* cluster. Several LmbU-like proteins in the BGCs also have been reported with positively regulatory effects. NovE activated the pathway-specific regulator gene *novG* for novobiocin biosynthesis [23, 24, 34]. And HmtD up-regulated the transcription of NRPS gene *hmtI* for himastatin biosynthesis at a fermentation time-dependent manner [26]. Different from those reported LmbU-like proteins, LmbU in *S. lincolnensis* acts as a positive cluster-scale regulator for lincomycin biosynthesis.

In addition to the *lmb* cluster, LmbU positively or negatively regulates the transcription of non-*lmb* genes in *S. lincolnensis*. 4-Hydroxyphenylpyruvate is precursor substrate for biosynthesis of l-tyrosine, which is hydroxylated by LmbB2 into l-dopa and initiates the biosynthesis of PPL moiety of lincomycin [9]. However, 4-hydroxyphenylpyruvate could be degraded by catalysis of HpdA [32] that was down-regulated by LmbU, thus it would lead to maintenance of l-tyrosine availability for PPL biosynthesis. In silico genomic sequence analysis of *S. lincolnensis* showed that the *hpdA* and *hpaR* (*SLCG_3525*) genes are clustered as the *hpd* operon. HpdR is an AsnC family transcriptional regulator and activates the *hpdA* transcription in the presence of 4-hydroxyphenylpyruvate and l-tyrosine [35]. In this study, the transcriptional level of *hpdR* was low and no obviously different between the original strain SyBE2901 and the lmbU deletion strain SyBE2904 (log_2_(FoldChange) = 0.55136), suggesting that the inhibitive effect of LmbU on the *hpdA* transcription might be HpdR-independent. It was interesting that LmbU not only represses the *hpdA* expression to increase l-tyrosine pool through inhibiting degradation of 4-hydroxyphenylpyruvate, but also promotes the expression of *lmbB1B2.* Both of them are benefit for PPL biosynthesis for l-tyrosine. Thus, LmbU plays at least a dual regulatory role at metabolic node of l-tyrosine as a substrate for lincomycin biosynthesis.

Absorptive ability of mycelia to the carbon and nitrogen sources from the fermentation medium is crucial for cell growth and lincomycin biosynthesis. LmbU facilitated the expression of the nitrate/nitrite transporter SLCG_3493 and sugar-binding protein SLCG_0089, which would force nitrate and sugar uptake and utilization. The similar function were reported on the nitrate transporters regulated by GlnR [16], suggesting that SLCG_3493 may be involved in lincomycin biosynthesis. SLCG_6997 similar to an oxalate:formate antiporter [36] and SLCG_1725 similar to threonine and homoserine efflux transporter [37] might be helpful for exchange of primary metabolites including organic acids and amino acids. Four proteins SLCG_7139, SLCG_7140, SLCG_7316, and SLCG_7561 could possess the multidrug efflux transport function. Especially, SLCG_7561 is a homolog of LmrA (48% of similarity in amino acid sequence), which also widely exists in lincomycin-resistant bacteria [38]. In addition to the resistance conferred by *lmrA*, *lmrB* and *lmrC* in the *lmb* cluster, the elevated expression of the non-*lmb* multidrug efflux transporter genes by up-regulation of LmbU might contribute higher self-resistance of *S. lincolnensis* to lincomycin and lead to the great amount of lincomycin production during the fermentation.

The steady-state redox potential is essential for primary and secondary metabolisms. Dehydrogenase SLCG_7376 might serve as a vital link between the tricarboxylic acid cycle and oxidative phosphorylation [39], and SLCG_2976, SLCG_6211 and SLCG_7612 would be involved in the reductive bioprocess [40]. SLCG_3218 functions as NADPH-dependent FMN reductase, and provides reducing power NADPH. LmbU up-regulates those oxidoreductase genes, and participates in cellular redox metabolisms.

Although majority of regulator genes in clusters are identified as pathway specific regulators of their cognate BGCs, we demonstrated that LmbU plays regulatory roles both in the *lmb* cluster and in non-*lmb* genes as aforementioned, exhibiting pleiotropic regulation. A few in-cluster regulators have been reported to have such regulatory effects on the BGCs and/or genes out of the BGCs. The PAS-LuxR family regulator FscRI within the candicidin BGC, not only activated genes in its cluster, but also provided a direct and essential regulation for expression of the antimycin BGC [41]. Moreover, apart from the cross-regulation of filipin and oligomycin biosynthesis, PteF in the pentaene filipin BGC could regulate DNA replication and repair, carbohydrate, lipid and energy metabolisms and morphological differentiation [42]. These results showed that the pleiotropic regulatory effects of the in-BGC regulators maybe not rare, and will be more frequently discovered following high throughput RNA-Seq technologies.

## Conclusions

The results of this study demonstrated that LmbU is a pleiotropic transcriptional regulator on lincomycin biosynthesis. In addition to direct activation of the whole *lmb* cluster, the regulatory effects of LmbU would be involved in l-tyrosine biosynthesis, nitrate and sugar transmembrane transport and utilization, multi-drug resistance, and oxidoreduction metabolisms by regulating the genes out of the *lmb* cluster. Our results provided evidence to elucidate the regulatory functions of LmbU-like proteins in actinomycetes.

## Methods

### Phylogenetic analyses of actinomycetes with LmbU-like proteins

LmbU-like proteins as were collected by BLASTP search against NCBI database. The flanking sequences of homologous *lmbU* genes (from upstream 50 kb to downstream 50 kb) were used to predict secondary metabolite BGCs by BLASTP and antiSMASH 5.0 [43]. The 16S rRNA genes of the host strains containing homologous LmbU proteins were used to construct phylogenetic tree alignments and phylogenetic analysis was performed using MEGA7 [44] by the neighbor-joining method (Kimura 2-parameter model + G) [45] and 500 bootstrap replications. The GenBank accession numbers of 16S sRNA genes and homologous LmbU proteins were listed in Additional file 1: Table S1.

### Strains, plasmids and culture conditions

The bacterial strains and plasmids used in this study are described in Table 1. Primers used in this study are listed in Additional file 1: Table S2. The sporulation and fermentation of *S. lincolnensis* strains were carried out as previously described [46]. Briefly, the spores of *S. lincolnensis* were routinely cultivated on the modified Gauze’s Medium No.1 (20 g l^−1^ soluble starch, 5 g l^−1^ soybean flour, 1 g l^−1^ KNO_3_, 0.5 g l^−1^ NaCl, 0.5 g l^−1^ MgSO_4_, 0.5 g l^−1^ K_2_HPO_4_, 0.01 g l^−1^ FeSO_4_, 18 g l^−1^ agar, pH 7.2 to 7.4) for 7 days at 30 °C. For shake flask fermentation, the spores inoculated into 25 ml of seed medium (20 g l^−1^ soluble starch, 10 g l^−1^ glucose, 10 g l^−1^ soybean, 30 g l^−1^ cream corn, 1.5 g l^−1^ (NH_4_)_2_SO_4_, 4 g l^−1^ CaCO_3_, pH 7.1) and grown for 2 days at 30 °C, 250 rpm. Then 2 ml of seed culture was added into a 250-ml flask containing 25 ml of fermentation medium (100 g l^−1^ glucose, 20 g l^−1^ soybean, 1.5 g l^−1^ cream corn, 8 g l^−1^ NaNO_3_, 5 g l^−1^ NaCl, 6 g l^−1^ (NH_4_)_2_SO_4_, 0.3 g l^−1^ K_2_HPO_4_, 8 g l^−1^ CaCO_3_, pH 7.1) and cultivated for 7 days at 30 °C and 250 rpm. Appropriate antibiotics were added in the medium when necessary.

### Inactivation and complementation of *lmbU*

For the *lmbU* in-frame deletion, the vector pAP07 was first constructed by the homologous recombination method. The 3.06-kb PCR fragment from the upstream (1105 bp) to the downstream (1275 bp) of *lmbU* was amplified from the genomic DNA of *S. lincolnensis* (isolated by Kirby mix procedure [48]) using primers DU-F and DU-R (Additional file 1: Table S2), digested by *Eco*R I, and cloned into SuperCos1 to yield vector pCosU. Using pIJ773 as template, the fragment *acc(3)IV*-*oriT* with 39-bp homologous sequences of *lmbU* at each terminus was amplified by primers QU-F and QU-R, and transformed into *E. coli* BW25113/pIJ790/pCosU. The *lmbU* gene on pCosU was replaced with *acc(3)IV*-*oriT* through the homologous recombination, resulting vector pAP07. To delete *lmbU*, the vector pAP07 was introduced into the original strain SyBE2901 by intergeneric conjugation from *E. coli* ET12567/pUZ8002/pAP07, following the procedure described previously [49]. The single-crossover mutants were identified with apramycin resistance, and then cultivated for several rounds without antibiotics. The double-crossover mutants for the *lmbU* deletion with kanamycin sensitivity and apramycin resistance were selected and confirmed by PCR using primers QU-F2 and QU-R2. Further sequencing verified that the 656-bp coding sequence of the *lmbU* gene on the genome was replaced with *acc(3)IV*-*oriT,* resulting the strain SyBE2904 with the in-frame deletion of *lmbU*.

For complementation of *lmbU*, the 1454-bp fragment containing the *lmbU* gene was amplified using 10U-F and 10U-R as primers, and ligated to pLCY010, pHZ1358, and pANT1201, resulting pLCY0010-U, pHZ1358-U and pANT1201-U, respectively. The 3245-bp fragment containing *lmbUYX* genes was amplified and ligated to pLCY010, resulting pLCY010-UYX. The vectors pLCY0010-U, pHZ1358-U pANT1201-U and pLCY010-UYX were introduced into the *lmbU* deletion strain SyBE2904, generating the complementation strain SyBE2929, SyBE2930, SyBE2931 and SyBE2913, respectively. The vector pLCY0010-U was introduced into the original strain SyBE2901, generating the *lmbU* overexpression strain SyBE2915.

### Analysis of lincomycin in fermentation broth

After cultivation, the supernatant was collected from fermentation broth (8000 rpm for 10 min), then mixed with 1.5 volumes of methanol and centrifuged again to remove the residue. The sample filtered through 0.22 μm of nylon membrane was subjected to HPLC analysis (Agilent 1200, USA) on C18 column (4.6 × 250 mm, 5 μm, Agela Technology, China) at UV 214 nm, 25 °C. The mobile phase was 50% ammonium acetate solution (0.005 mol l^−1^, pH 9.0) and 50% methanol. The concentration of lincomycin was estimated by HPLC using the lincomycin standard (National Institute for the Control of Pharmaceutical and Biological Products, China).

### RNA-Seq transcriptomic analysis

Because of the complexity of bacterial transcriptome, we used strand specific RNA sequencing (ssRNA-Seq) to investigate transcription of *S. lincolnensis*. The total RNA was extracted from *S. lincolnensis* after growth for 3 days. To enrich mRNA, rRNA was removed by Ribo-zero kit. The strand-specific libraries for high throughput RNA sequencing were generated using NEBNext Ultra Directional RNA Library Prep Kit for Illumia (NEB). Then fragmentation buffers were added to break the mRNA into short fragments. The 1st cDNA was synthesized with random primers using the mRNA as template. The buffer solution, dNTPs (dTTP was replaced by dUTP), DNA polymerase I and RNase H were added to synthesize the double-stranded cDNA. After been purified by AMPure XP beads, the double-stranded cDNAs were repaired at the end, attached with the A-tail, ligated with Y-shape adapter and selected with the fragment size by AMPure XP beads. And then the “U” containing strand of the adapter ligated DNA was degraded by USER-enzyme. Finally, the strand-specific libraries were enriched by PCR amplification, and then deep sequenced on an Illumina Hiseq 2500 platform. The raw sequence data were filtered by removing reads containing adapter, reads containing more than 10% N (undetermined base), and low-quality reads (reads containing more than 50% bases with mass value Qpred < 5). The clean reads were aligned with the genome of *S. lincolnensis* LC-G (GenBank ID: 1435096411) by Bowtie2-2.2.3 [52]. The number of FPKM (expected number of Fragments Per Kilobase of transcript sequence per Millions base pairs sequenced) was used to quantify gene expression. Genes with |log2(FoldChange)| > 1 and *p* value < 0.005 found by DEGSeq were assigned as differentially expressed.

### Semi-quantitative PCR analysis

For RNA extraction, mycelia of *S. lincolnensis* original strain SyBE2901 and the *lmbU* deletion strain SyBE2904 were collected from 25-ml culture (3 days of growth in fermentation medium) by centrifugation, and ground into powder in liquid nitrogen. Total RNA was extracted using RNApure kit (Bio Teke, China) according to the manufacturer’s protocol.

Reverse transcription was conducted using TransScript II One-Step gDNA Removal and cDNA Synthesis SuperMix kit (TransGene Biotech, China). Using gene specific primers (reverse primer used in semi-quantitative PCR) and 500 ng of total RNA as template, the first strand was generated. For analyzing transcription, the 1st cDNA reaction mixture was used as the template to amplify ds-cDNA in the following semi-quantitative PCR. In each case the experiments were conducted in triplicate. The PCR products were detected using agarose gel electrophoresis, and then exposed under UV to analyze relative intensity. Primers used in semi-quantitative PCR experiments were listed in Additional file 1: Table S2.

## Supplementary information

**Additional file 1: Fig. S1.** Semi-quantitative PCR of the non-*lmb* genes in strains SyBE2901 and SyBE2904 for 30 and 34 thermocycles, respectively. In each case the experiments were repeated three times. **Table S1.** The actinomycetes with 16S rRNA genes and LmbU-like proteins used for constructing the phylogenetic trees in Fig. 1. **Table S2.** Primers used in this study.

## Data Availability

The datasets used and/or analyzed during the current study are available from the corresponding author on reasonable request.

## Abbreviations

BGC: Biosynthetic gene cluster
*lmb* cluster: Lincomycin biosynthetic gene cluster
ORF: Open reading frames
ssRNA-Seq: Strand specific RNA sequencing
TSS: Transcription start site
TTS: Transcription termination site
TU: Transcription unit
RT-PCR: Reverse transcription polymerase chain reaction
ds-cDNA: Double-stranded cDNA
1st cDNA: First strand DNA
MFS transporter: Major facilitator superfamily transporter
ABC: ATP-binding cassette
DMT: Drug/metabolite transporter
MTL: Methylthiolincosaminide
PPL: 4-Propyl-l-proline
LSM: Lincosamide
EGT: Ergothioneine
