# Supplementary material for: Comparative transcriptomic analysis reveals the significant pleiotropic regulatory effects of LmbU on lincomycin biosynthesis
Source: Microb Cell Fact. 2020 Feb 12;19:30. doi: 10.1186/s12934-020-01298-0 (PMC7014725; doi:10.1186/s12934-020-01298-0)
Supplement: Supplementary file 1 — Additional file 1: Fig. S1. Semi-quantitative PCR of the non-lmb genes in strains SyBE2901 and SyBE2904 for 30 and 34 thermocycles, respectively. In each case the experiments were repeated three times. Table S1. The actinomycetes with 16S rRNA genes and LmbU-like proteins used for constructing the phylogenetic trees in Fig. 1. Table S2. Primers used in this study. [file 12934_2020_1298_MOESM1_ESM.docx]

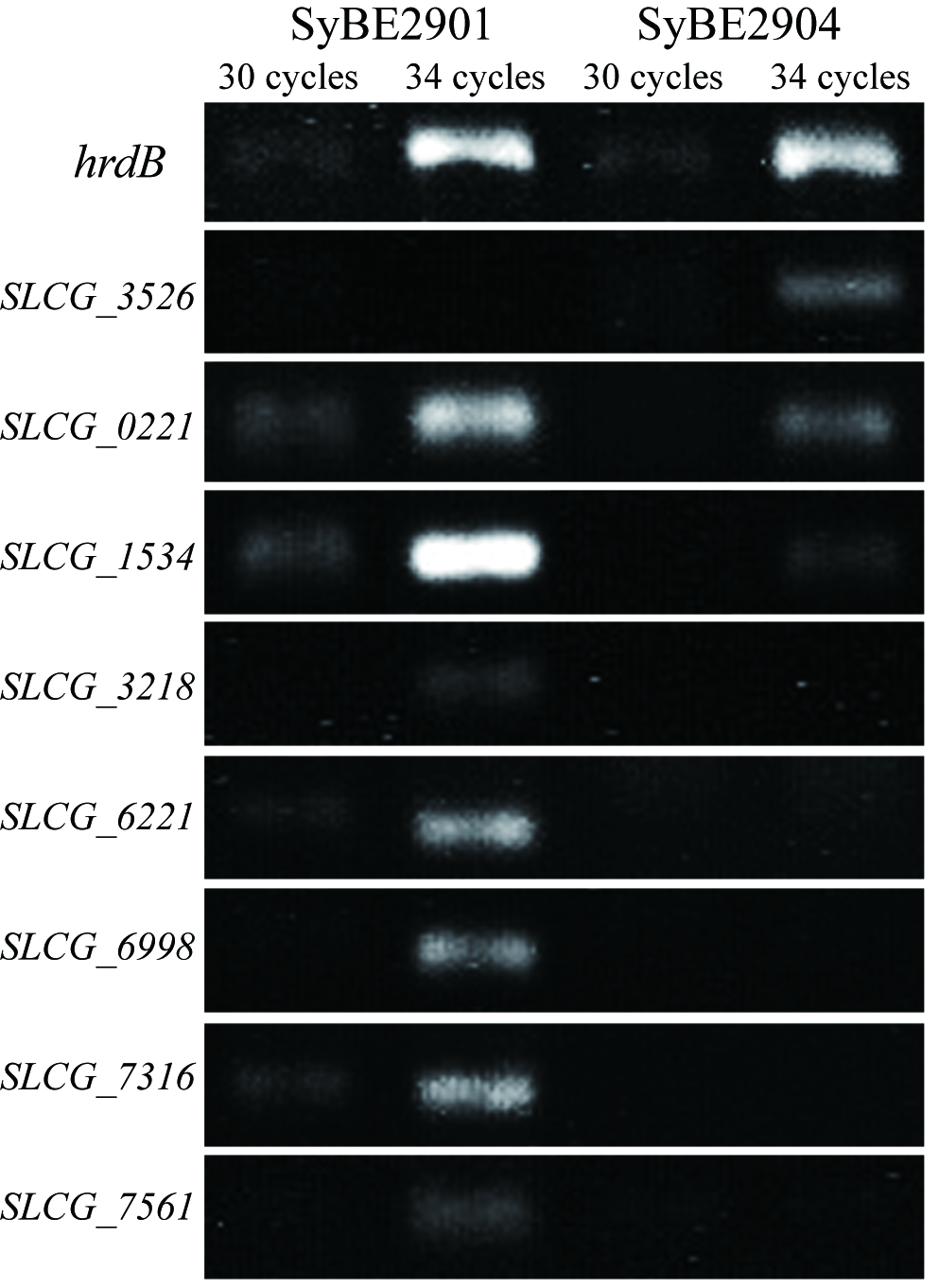


**Fig. S1** Semi-quantitative PCR of the non-*lmb* genes in strains SyBE2901 and SyBE2904 for 30 and 34 thermocycles, respectively. In each case the experiments were repeated three times.

**Table S1** The actinomycetes with 16S rRNA genes and LmbU-like proteins used for constructing the phylogenetic trees in **Fig. 2.**

| Species | Accession number of 16S rRNA gene | LmbU-like protein(s) |
| --- | --- | --- |
| *Streptomyces lincolnensis* LC-G | CP022744 | AXG51408.1 |
| *Streptomyces violaceoruber* S21 | CP020570.1 | ARF66340.1 |
| *Streptomyces* sp. W007 | JN180126.1 | EHM26079.1 |
| *Streptomyces griseus* subsp. *rhodochrous* | LGUZ01000077.1 | KOG76434.1 |
| *Streptomyces* sp. NEAU-D10 | QUAC01000041.1 | REK87017.1 |
| *Amycolatopsis japonica* MG417-CF17 | CP008953.1 | AIG79897.1 |
| *Saccharopolyspora erythraea* NRRL 2338 | PDBV01000001.1 | PFG98425.1 |
| *Streptomyces lydicus* 103 | CP017157.1 | AOP51509.1 |
| *Frankia alni* ACN14a | CT573213.2 | CAJ63269.1 CAJ61184.1 CAJ61188.1 |
| *Streptomyces griseofuscus* NG1-7 | MTKC01000001.1 | OSP07050.1 |
| *Kutzneria buriramensis* DSM 45791 | QUNO01000051.1 | REH43559.1 REH46913.1 |
| *Umezawaea tangerina* DSM 44720 | PVTF01000029.1 | PRY36961.1 |
| *Saccharothrix espanaensis* DSM 44229 | HE804045.1 | CCH33122.1 |
| *Streptomyces anulatus* ATCC 11523 | JPZP01000006.1 | ADG27350.1 ADG27365.1 |
| *Actinophytocola xinjiangensis*  CGMCC 4.4663 | MSIF01000056.1 | OLF07425.1 |
| *Saccharothrix carnea*  CGMCC 4.7097 | PYAX01000037.1 | PSL53083.1 |
| *Streptomyces regensis* strain NRRL B-11479 | LFVR01000689.1 | KMS78307.1 |
| *Streptomyces luteus* TRM 45540 | JNFQ01000003.1 | KFG71222.1 |
| *Streptomyces parvulus* 2297 | CP015866.1 | ANJ11297.1 ANJ11944.1 |
| *Streptomyces curacoi* DSM 40107 | LMWJ01000045.1 | KUM75147.1 |
| *Saccharothrix texasensis* DSM 44231 | RJKM01000001.1 | ROP41278.1 |
| *Streptomyces phaeoluteigriseus* DSM 41896 | MPOH02000009.1 | OQD55160.1 OQD55161.1 OQD51816.1 |
| *Amycolatopsis coloradensis* DSM 44225 | MQUQ01000038.1 | OLZ54755.1 OLZ55751.1 |
| *Streptomyces resistomycificus* DSM 40133 | LMWZ01000052.1 | KUN90612.1 KUN90566.1 |
| *Amycolatopsis decaplanina* DSM 44594 | NR_025562.1 | EME57734.1 |
| *Nocardiopsaceae bacterium* YIM 96095 | RJMB01000020.1 | RNL86219.1 RNL80540.1 |
| *Streptomyces himastatinicus* ATCC 53653 | NR_044201.1 | CBZ42138.1 |
| *Streptomyces lunaelactis* MM109 | CP026304 | AVZ77915.1 AVZ77431.1 |
| *Lentzea flaviverrucosa* CGMCC 4.578 | FOFT01000032.1 | SER83110.1 |
| *Streptomyces antibioticus* DSM 41481 | LHQL01000006.1 | OOQ48465.1 |
| *Kutzneria* sp. 744 | DQ181633.1 | ABV56606.1 |
| *Lentzea jiangxiensis* | FNIX01000061.1 | SDP94264.1 |
| *Amycolatopsis sulphurea* DSM 46092 | NR_114439.1 | PFG57269.1 PFG49148.1 |
| *Streptomyces yanglinensis* CGMCC 4.2023 | FNVU01000044.1 | SEF53484.1 SEF67665.1 SEF52576.1 |
| *Sinosporangium album* CPCC 201354 | FNCN01000084 | SDH07190.1 SDH93941.1 |
| *Streptomyces alboflavus* MDJK44 | CP021748.1 | ARX87956.1 ARX88788.1 |
| *Streptomyces puniciscabiei* TW1S1 | CP017248.1 | AOR37027.1 |
| *Streptomyces silvensis* ATCC 53525 | NZ_LOCL01000062.1 | KUF15258.1 KUF16388.1 |
| *Streptomyces malaysiense* MUSC 136 | LBDA02000093.1 | OIK28211.1 |
| *Kibdelosporangium phytohabitans* KLBMP1111 | CP012752.1 | ALG14998.1 ALG14996.1 |
| *Streptomyces hyalinus* NBRC 13850 | LC436365.1 | GCD93473.1 |
| *Nocardiopsis alba* ATCC BAA-2165 | CP003788.1 | AFR08057.1 |
| *Streptomyces albulus* B-3066 | LWBU01000148.1 | OAL11542.1 |
| *Actinomadura madurae*  DSM 43067 | NR_026343.1 | SFO39606.1 |
| *Nocardiopsis dassonvillei*  NOCA502F | CP017965.1 | APC38596.1 |
| *Streptomyces fungicidicus* TXX3120 | CP023407.1 | AYL35094.1 |
| *Microbispora rosea* ATCC 12950 | FTNI01000083.1 | SIQ19893.1 |
| *Streptomyces griseochromogenes* ATCC 14511, | CP016279.1 | ANP56432.1 ANP56462.1 |
| *Streptomyces tsukubensis* F601 | MVFC01000096.1 | OON71554.1 |
| *Streptomyces rapamycinicus* NRRL 5491 | QYCY01000001.1 | AGP53450.1 |
| *Saccharomonospora glauca* K62 | AGJI02000002.1 | EIE98960.1 |
| *Nocardiopsis gilva* YIM 90087 | CP022753.1 | ASU81751.1 |
| *Streptomyces clavuligerus* ATCC 27064 | CM000913.1 | EFG10704.1 |
| *Streptomyces atratus* OK807 | FPJO01000093.1 | SFX77725.1 |
| *Actinosynnema pretiosum*  ATCC 31280 | CP029607.1 | AXX31942.1 |
| *Streptomyces iranensis* | NZ_LK022848.1 | CDR04912.1 |
| *Streptomyces antioxidans* MUSC 164 | LAKD02000007.1 | OPF82238.1 |
| *Micromonospora rifamycinica* DSM 44983 | LT607752.1 | SCG59159.1 |
| *Streptomyces albus* subsp. *albus* NRRL F-4371 | LMZE01000109.1 | KWT63939.1 RKT54708.1 |
| *Saccharothrix australiensis* strain DSM 43800 | RBXO01000001 | RKT54687.1 RKT54708.1 |
| *Streptomyces katrae* NRRL ISP-5550 | JZWV01000648.1 | KJY37858.1 |
| *Solirubrobacter pauli* DSM 14954 | RBIL01000002.1 | RKQ84961.1 RKQ86780.1 |
| *Streptomyces afghaniensis* 772 | AOPY01001400.1 | EPJ34270.1 |
| *Streptomyces chartreusis* NRRL 3882 | NZ_LT962942.1 | SOR78108.1 |
| *Kibdelosporangium aridum* strain A82846 | QHKI01000152.1 | RSM82015.1 |
| *Actinoalloteichus hoggarensis* DSM 45943 | CP022521.1 | ASO20621.1 |
| *Gaiella occulta* | QQZY01000002.1 | RDI75853.1 |
| *Nonomuraea jiangxiensis* CGMCC 4.6533 | FNDJ01000065.1 | SDM82708.1 |
| *Streptomyces yokosukanensis* strain DSM 40224 | LMWN01000153.1 | KUM99164.1 |
| *Lentzea aerocolonigenes* NRRL B-16140 | JYJG01000143.1 | KJK33586.1 |
| *Streptomyces aurantiacus* JA 4570 | AOPZ01000103.1 | EPH46592.1 |
| *Nonomuraea solani* CGMCC 4.7037 | FNVT01000073.1 | SEH03093.1 |
| *Couchioplanes caeruleus* DSM 43634 | RJKL01000001.1 | ROP31312.1 |
| *Escherichia coli* K-12 | LN832404.1 |  |

**Table S2** Primers used in this study

| Primers | Sequence (5’-3’) |
| --- | --- |
| Construction of the *lmbU* overexpression and inactivation | |
| ΔU-F | GCCGGAATTCGGTACCCAGCGAGGAATGAC |
| ΔU-R | GCCGGAATTCAGCGTCCTGGACAACCTGC |
| QU-F | **GAGAACCAGCGACGCGGCGAAGTCGAGCCGGGCGCAAAT**ATTCCGGGGATCCGTCGACC |
| QU-R | **GCTGCTTGCTTTCGATCCCCGTGTCCTGCTCGGTGACCC**TGTAGGCTGGAGCTGCTTC |
| QU-F2 | GGATGTTGGCGACGAGGGT |
| QU-R2 | TTGGGTTGCCGCTTTGGAT |
| 1201U-F | GCCCAAGCTTAGCTTGCCCCGGATTTGT |
| 1201U-R | GCCGGAATTCGCGTACCGCCATAATTCACG |
| 1358U-F | CTAGTCTAGATTGGGTTGCCGCTTTGGA |
| 1358U-R | CTAGTCTAGAGACCTCTTTGGCGAACACGAC |
| 010U-F | GCCCAAGCTTAGCTTGCCCCGGATTTGT |
| 010U-R | GCCGGAATTCGCGTACCGCCATAATTCACG |
| 010UYX-F | GCCCAAGCTT AGCTTGCCCCGGATTTGT |
| 010UYX-R | CCAGTCTAGA GGTGAATCGGTGACGGTGAAT |
| Semi-quantitative RT-PCR For the *lmb* gene | |
| rA-rtF | AAGGCGTTGAGCGTCTGGG |
| rA-rtR | TGGTCAGCAGGAAGAAGGAGGT |
| A-rtF | ATGCGGGCATGAGAATCCTTG |
| A-rtR | GCCAGCGGTATGTCGGTGAA |
| B2-rtF | AGGCGAATCAGTTTCAGCACG |
| B2-rtR | CACGGTGAGGAAGACGCAGTC |
| C-rtF | ACCTTGAGGACGGTCGTGTTCG |
| C-rtR | CCACCTTCACCACGCAGCAGT |
| D-rtF | GCGAACGTGGCGAAGAGGGT |
| D-rtR | GCGTCGATGTCGTGCAACAGG |
| E-rtF | ATGACTCAGTGCCTGCTGACCG |
| E-rtR | CGAGAAGGCGATGTGGTAGACG |
| F-rtF | CCGACGACGGCTGGCTGAT |
| F-rtR | TGTGCTGCGGGTTGATGAGG |
| G-rtF | CCTGCACGGCCTCGACAACT |
| G-rtR | AGGGCCTCCGGGTCCATCTT |
| J-rtF | GCGAAGCTCTACGGTGACAAGA |
| J-rtR | GCACTGAAGGACCAGGAGGC |
| K-rtF | GGACGCGAGGGACAGTCGA |
| K-rtR | CGTGGAGGCAGGTGAAGAAGGA |
| L-rtF | CACCGCACGTTCGCTCTTCC |
| L-rtR | CGGTCCTTCAGGTTCCCGAGTT |
| M-rtF | CCTCAGCAACACCACCACCC |
| M-rtR | GATGCCGTCCACGCACTCCT |
| N-rtF | CGGCGACGAGACCGAACTCA |
| N-rtR | GGTGGCGTGCGGGATGTTGA |
| Z-rtF | TCGATCTGCTGCGGAGCTACTT |
| Z-rtR | CGGTTTCTCCCAGGTGAGGGT |
| O-rtF | CACCGATGTCGTGCTGTGCA |
| O-rtR | CGCCGTAGAAGTCCGCCTC |
| R-rtF | TCCGTGGTGGAACAGTGGCT |
| R-rtR | GGTCATCACGTCGAGGTGGG |
| T-rtF | GCGGTTCTTCCACGCCCTGT |
| T-rtR | GCCACCCACATCGCCTCCAT |
| W-rtF | TCGGCAAAGCCCGGAAAT |
| W-rtR | TGCTCGCCATGACGGAAGT |
| rB-rtF | CCCTGACGCTGCCCTTGAGT |
| rB-rtR | CGCTTGAGCACCGAGAAGTCG |
| X-rtF | GTTCGCCCACGAGCCCTTCT |
| X-rtR | GGTGCCCTTGTCCGGTCCTA |
| Y-rtF | ACCAGCCATCACCTCCACCC |
| Y-rtR | GCCCATCGTGACCCAGGTGT |
| U-rtF | TGGTGTCTGGGCGACTGGA |
| U-rtR | GGCGGGTGTCGGACTTCTT |
| rC-rtF | GCCTCTACGGTGGCAACTTCA |
| rC-rtR | CCTCATGGCACGTCAACACG |
| Semi-quantitative RT-PCR for non-*lmb* gene | |
| SLCG_3526F | CGAGTACAGCGCCCTGATG |
| SLCG_3526R | GCCTTGAAGTTGCCCTTGC |
| SLCG_0221F | TTTCGTGGCCGAGGAGCA |
| SLCG_0221R | TGACGGTCGCCTCATACGC |
| SLCG_1534F | TGGAGGTCAACCATGCCGAGAC |
| SLCG_1534R | GGAAGAAGCAGCGGGAGACGA |
| SLCG_3218F | GACATGGAGGTCCACGTCATCG |
| SLCG_3218R | CAGGCGAGTTGGTCCAGCAGT |
| SLCG_6211F | TCGCAGTGGAGGGACGAGC |
| SLCG_6211R | CGTCGGTGCTGAGGTGGAAG |
| SLCG_6997F | CGATGAAGCGGCTGATGC |
| SLCG_6997R | AAGTCGTGCCACGTCGAGTAG |
| SLCG_7316F | GTCCTGCACGGCATCTCCTTC |
| SLCG_7316R | CGAGGTGTTGTCGAGGTGGG |
| SLCG_7561F | CGAACCTTGCCCAGGCGTAT |
| SLCG_7561R | TCCCACCAGATGAAGAGCACC |
